# Spatio-temporal analysis of the relationship between meteorological factors and hand-foot-mouth disease in Beijing, China

**DOI:** 10.1186/s12879-018-3071-3

**Published:** 2018-04-03

**Authors:** Lin Tian, Fengchao Liang, Meimei Xu, Lei Jia, Xiaochuan Pan, Archie C. A. Clements

**Affiliations:** 1 Department of Occupational and Environmental Health, School of Public Health, Peking University, Beijing, 100191 China; 2 Department of Epidemiology, Fuwai Hospital, State Key Laboratory of Cardiovascular Disease, National Center for Cardiovascular Diseases, Chinese Academy of Medical Sciences and Peking Union Medical College, Beijing, 100037 China; 3 Institute of Medical Information, Chinese Academy of Medical Sciences and Peking Union Medical College, Beijing, 100020 China; 4 Institute for Infectious Disease and Endemic Disease Control, Beijing Center for Disease Prevention and Control, Beijing, 100013 China; 5 Research School of Population Health, College of Medicine, Biology and Environment, The Australian National University, Canberra, ACT Australia

**Keywords:** Hand-foot-mouth disease, Spatio-temporal analysis, Bayesian approach, Meteorological factors

## Abstract

**Background:**

Hand-foot-mouth disease (HFMD) is a common infectious disease in China and occurs mostly in infants and children. Beijing is a densely populated megacity, in which HFMD has been increasing in the last decade. The aim of this study was to quantify spatio-temporal characteristics of HFMD and the relationship between meteorological factors and HFMD incidence in Beijing, China.

**Methods:**

Daily counts of HFMD cases from January 2010 to December 2012 were obtained from the Beijing Center for Disease Prevention and Control (CDC). Seasonal trend decomposition with Loess smoothing was used to explore seasonal patterns and temporal trends of HFMD. Bayesian spatiotemporal Poisson regression models were used to quantify spatiotemporal patterns of HFMD incidence and associations with meteorological factors.

**Results:**

There were 114,777 HFMD cases reported to Beijing CDC from 1 January 2010 to 31 December 2012 and the raw incidence was 568.6 per 100,000 people. May to July was the peak period of HFMD incidence each year. Low-incidence townships were clustered in central, northeast and southwest regions of Beijing. Mean temperature, relative humidity, wind velocity and sunshine hours were all positively associated with HFMD. The effect of wind velocity was significant with a RR of 3.30 (95%CI: 2.37, 4.60) per meter per second increase, as was sunshine hours with a RR of 1.20 (95%CI: 1.02, 1.40) per 1 hour increase.

**Conclusions:**

The distribution of HFMD in Beijing was spatiotemporally heterogeneous, and was associated with meteorological factors. Meteorological monitoring could be incorporated into prediction and surveillance of HFMD in Beijing.

**Electronic supplementary material:**

The online version of this article (10.1186/s12879-018-3071-3) contains supplementary material, which is available to authorized users.

## Background

Hand, foot and mouth disease (HFMD) is a common infectious disease that occurs mostly in infants and children, but can also occur in adolescents and occasionally in adults. HFMD is mainly caused by coxsackievirus A16 (Cox A16), which usually results in a mild self-limiting disease with few complications, and enterovirus 71 (EV71), which has been associated with serious complications and may be fatal. [1, 2] Outbreaks of HFMD have occurred in the Western Pacific Region in recent years, and China is one of the Asian countries with the highest number of reported cases [2, 3]. An EV71 vaccine was first generated and approved in China in 2016. However, several recent HFMD outbreaks have been caused by other pathogens, making it important to develop multivalent vaccines to control HFMD epidemics [4].

HFMD has been listed as a notifiable Class C infectious disease in the national communicable disease surveillance and reporting system of China since May 2008. Compared with 2008, the number of reported cases in 2010 was approximately four times higher [5]. In January 2017, there were 77,412 cases of HFMD including 5 deaths reported in mainland China [6]. The increasing burden of HFMD has become a serious public health problem in China, causing government concern about the risk factors of this disease and greater interest in approaches to effectively prevent and control HFMD.

In China, the epidemiological characteristics, risk factors and spatiotemporal patterns of HFMD have been studied at a national scale [3, 7–10]. Considering the extensive area and diverse demographic, economic and climatic characteristics of China, the distribution and risk factors likely vary in different regions. Previous studies in Guangzhou, Wuhan, Zhengzhou, Shanxi and Hong Kong have explored relationships between meteorological variables and HFMD using time-series analysis, through which the seasonal and temporal characteristics of HFMD have been characterized. Temperature and relative humidity were found to be associated with incidence of this disease [11–16]. In Beijing, Xu et al. [17] found a non-linear association between temperature and HFMD incidence.

In addition to temporal dynamics, HFMD epidemics demonstrate spatial patterns, which have been characterized in different areas [18–22]. However, few spatio-temporal studies have explored the relationship between HFMD incidence and meteorological factors and simultaneously quantified spatial and temporal patterns. Bayesian approaches could be utilized both to quantify spatio-temporal variation and identify how meteorological factors affect HFMD incidence [23, 24]. Such studies could help establish the utility of meteorological monitoring as an adjunct for the surveillance and control of HFMD.

This study aims to quantify seasonal patterns, temporal trends and the spatio-temporal distribution of HFMD incidence at a township level in Beijing, and investigate the relationship between meteorological factors and HFMD incidence in this city.

## Methods

### Study area

Beijing, the capital city of China, is located in the northern tip of the roughly triangular North China Plain, at a latitude of 39″26′ to 41″03’ N. The area of Beijing is 16,410 km^2^ with 14 urban administrative districts and two rural counties, comprised of 304 townships. The population is 19.6 million, with townships ranging from 2000 to 359,400 people (The Sixth National Population Census, Beijing, 2010). Beijing has a rather dry, monsoon-influenced continental climate with hot, humid summers and cold, dry winters. As townships are the smallest administrative units for monitoring of infectious diseases, they were used as the geographical unit for this spatiotemporal analysis.

### HFMD data

Data on HFMD cases that were reported from January 2010 to December 2012 were obtained from the Beijing Center for Disease Prevention and Control (CDC). The data were collected from the China Information System for Disease Control and Prevention (CISDCP) with age, gender, occupation, address and dates of onset and diagnosis. HFMD cases were diagnosed based on the symptoms of fever, vesicular lesions on hands, feet, mouth and occasionally the buttocks, which are defined by the National Guidelines published by Chinese Ministry of Health in 2009 [25]. Severe cases are HFMD cases associated with meningitis, encephalitis, and severe complications, including neurological, cardiovascular and respiratory problems. All HFMD cases are required to be reported to CISDCP within 24 h after diagnosis.

### Meteorological and population data

Meteorological factors investigated included mean temperature, maximum temperature, minimum temperature, relative humidity, atmospheric pressure, precipitation, sunshine hours and wind velocity. Daily data on these variables were collected from the China Meteorological Data Sharing Service System (http://data.cma.cn/). A weather monitoring station is located in Daxing District (N39°48′, E116°28′) in southeast Beijing, from which the data are usually used to represent the meteorological conditions of the whole of Beijing city [17]. Thus, the meteorological data were temporally but not spatially variable. We obtained population data from the Beijing Area Statistics Yearbook from 2005 to 2015, which were used to calculate linear monthly growth rates at the township level and to estimate monthly population counts between January 2010 and December 2012.

### Statistical analysis

Seasonal trend decomposition with Loess smoothing was used to explore seasonal patterns and temporal trends of HFMD, using the statistical software R version 3.3.2 (R Development Core Team, 2016).

Pairwise Spearman correlation analysis was conducted to detect correlations between the meteorological factors. For pairs of variables with a correlation coefficient > |0.7|, only one member of the pair was included in multivariable models (the member with the lowest *p*-value in a bivariate regression model).

The standardized morbidity ratios (SMRs) of each town were calculated by dividing the observed number of cases by the expected number, which was calculated as the product of overall incidence of the city and the average population for each township during the study period.

In this study we used a Bayesian conditional auto regressive (CAR) model approach, with parameter estimation done by Markov chain Monte Carlo (MCMC) methods. This approach allows estimation of spatial variability of disease risk and the effect of covariates [26, 27], overcoming the issue of spatial autocorrelation violating the assumption of independence [23, 28]. In recent years, CAR models have been used to analyze the spatial distribution of malaria, filariasis, and schistosomiasis [26, 29, 30], but there have been fewer applications to other infectious diseases.

Using the Bayesian framework, Poisson regression models were used to quantify spatiotemporal variation of HFMD incidence and associations with selected meteorological factors. The models were implemented using the WinBUGS software, version 1.4.3 (Medical Research Council, Biostatistics Unit, Cambridge, United Kingdom). Three Bayesian models (non-spatial, spatial and spatiotemporal) were constructed. The regression models assumed that the observed HFMD cases followed a Poisson distribution with a mean (*μ*):

$$ {Y}_{ij}\sim Poisson\left({\mu}_{ij}\right) $$

$$ L\mathrm{og}\left({\mu}_{ij}\right)= Log\left({E}_{\mathrm{ij}}\right)+{\theta}_{\mathrm{ij}}, $$

where *Y*_*ij*_ is the observed number of cases in town *i*, month *j*; *E*_*ij*_ is the expected number of cases in town *i*, month *j*; and *θ*_ij_ is the relative risk (SMR) in town *i*, month *j*.

The non-spatial model was defined as:

$$ {\theta}_{ij}=\alpha +\sum \limits_k{\beta}_k{X}_{kij}+{v}_i+{g}_j+{\omega}^{\ast }{month}_j $$

The spatial model was defined as:

$$ {\theta}_{ij}=\alpha +\sum \limits_k{\beta}_k{X}_{kij}+{v}_i+{u}_i+{g}_j+{\omega}^{\ast }{month}_j $$

The spatio-temporal model was defined as:

$$ {\theta}_{ij}=\alpha +\sum \limits_k{\beta}_k{X}_{kij}+{v}_i+{g}_j+{u}_i+{d_i}^{\ast }{month}_{ij}+{\omega}^{\ast }{month}_j, $$

where *α* is the intercept; *β*_*k*_ are the k regression coefficients and *X*_*kij*_ are the covariates (different meteorological factors); *v*_*i*_ is an unstructured random effect with mean zero and variance ${\sigma}_v^2$; *u*_*i*_ is a spatially structured random effect with mean zero and variance ${\sigma}_u^2$; *g*_*j*_ is the autoregressive time effect with mean zero and variance ${\sigma}_g^2$; *d*_*i*_ are the spatially smoothed town-level temporal trend coefficients; and *ω* is the provincial average temporal trend coefficient.

For the intercept (α) flat prior distributions was applied. For all the coefficients (β) and the provincial average temporal trend coefficient (*ω*), normal prior probability distributions were used and assumed with a mean = 0 and a precision (inverse of variance) = 0.0001. The spatial structuring in structured random effect (*u*_*i*_) and spatially smoothed town-level temporal trend coefficients (*d*_*i*_) was modelled using a CAR prior structure, which is defined by a simple adjacency weights matrix to determine the spatial relationships between townships. If two towns were adjacent, the weight = 1 and if they were not adjacent, the weight = 0. The weights matrix was generated in ArcGIS (version 10.3, ESRI, Redlands, CA). The priors for the precision of *v*_*i*_, *u*_*i*_, *g*_*j*_ and *d*_*i*_ were specified using non-informative gamma distributions with a shape parameter = 0.5 and a scale parameter = 0.0005.

The model with the lowest deviance information criterion (DIC) was chosen as the best-fitting model. The model was run for 300,000 iterations after an initial burn-in of 10,000 iterations, and was assessed for convergence. After model convergence, samples from the posterior distributions of each random variable were stored and analyzed to provide summary estimates.

## Results

### Seasonal patterns and temporal trends of HFMD

There were 114,777 HFMD cases reported to Beijing CDC from 1 January 2010 to 31 December 2012, of which the sex ratio was 1.5 (male/female). There were 1244 (1.08%) severe cases and 27 (0.02%) deaths. Five thousand eight hundred and ninety-one cases were laboratory confirmed with 2516 infected with Cox A16 (42.71%) and 2406 infected with EV17 (40.84%). Most cases were distributed in children aged 1 to 4 years old (78.88%). Of all the children who were reported to have HFMD, 51.29% were cared for in homes, with an incidence of 292.7 per 100,000 people from 2010 to 2012, and 42.63% were cared for in nurseries, with an incidence of 243.3 per 100,000 people from 2010 to 2012 (Table 1). Table 2 shows the Spearman correlation of meteorological factors. Mean temperature, relative humidity, precipitation, wind velocity and sunshine hours were included in multivariable models for pairs of these variables with correlation coefficients less than |0.7|.

**Table 1:** Summary characteristics of HFMD cases in Beijing, 2010–2012

	2010			2011			2012			Total
Age	Male	Female	Total	Male	Female	Total	Male	Female	Total	
0-	1606	972	2578	1081	610	1691	1055	722	1777	6046
1-	5819	3770	9589	3823	2383	6206	4409	3070	7479	23,274
2-	5851	3888	9739	3882	2710	6592	4460	2884	7344	23,675
3-	6505	4187	10,692	4310	2906	7216	5423	3426	8849	26,757
4-	3674	2408	6082	2775	1849	4624	3684	2445	6129	16,835
5-	1918	1332	3250	1279	834	2113	1967	1297	3264	8627
6–14	1745	1166	2911	1168	861	2029	1869	1335	3204	8144
≥15	253	313	566	155	216	371	212	270	482	1419
Occupation										
Scattered children	14,246	9226	23,472	9415	6147	15,562	11,938	7902	19,840	58,874
Nursery children	11,688	7642	19,330	8128	5383	13,511	9725	6365	16,090	48,931
Others	1437	1168	2605	930	839	1769	1412	1182	2594	6968
Pathogen										
Cox A16	371	255	626	361	246	607	787	496	1283	2516
EV17	583	405	988	351	243	594	488	336	824	2406
Other	281	166	447	122	81	203	193	126	319	969
Number of severe cases	378	232	610	175	103	278	215	141	356	1244
Number of deaths	14	4	18	4	1	5	3	1	4	27
Total	27,371	18,036	45,407	18,473	12,369	30,842	23,079	15,449	38,528	114,777

**Table 2:** Pairwise Spearman correlation of meteorological factors

	Meant	Maxt	Mint	rh	ap	Rain	ws
Maxt	0.987*						
Mint	0.981*	0.947*					
rh	0.389*	0.334*	0.474*				
ap	−0.869*	−0.865*	−0.851*	−0.375*			
Rain	0.181*	0.142*	0.259*	0.471*	−0.248*		
ws	−0.009	−0.015	−0.003	−0.448*	−0.02	0.033	
sun	0.165*	0.248*	0.032	−0.571*	−0.072*	−0.339*	0.273*

*Meant* mean temperature, *maxt* maximum temperature, *mint* minimum temperature, *rh* relative humidity, *ap* atmospheric pressure, *rain* precipitation, *ws* wind velocity, *sun* sunshine hours

*P < 0.05

**Fig. 1:**
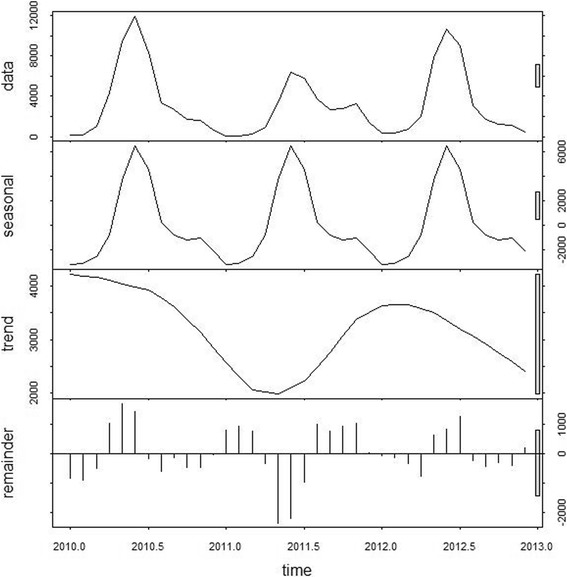
Decomposed HFMD time-series in Beijing, 2010–2012

Figure 1 shows the time-series decompositions of HFMD cases. There was an evident seasonal pattern with a peak occurring between May and July of each year. After the seasonal pattern was removed, the inter-annual pattern was characterized by peaks at the start and towards the end of the study period.

**Fig. 2:**
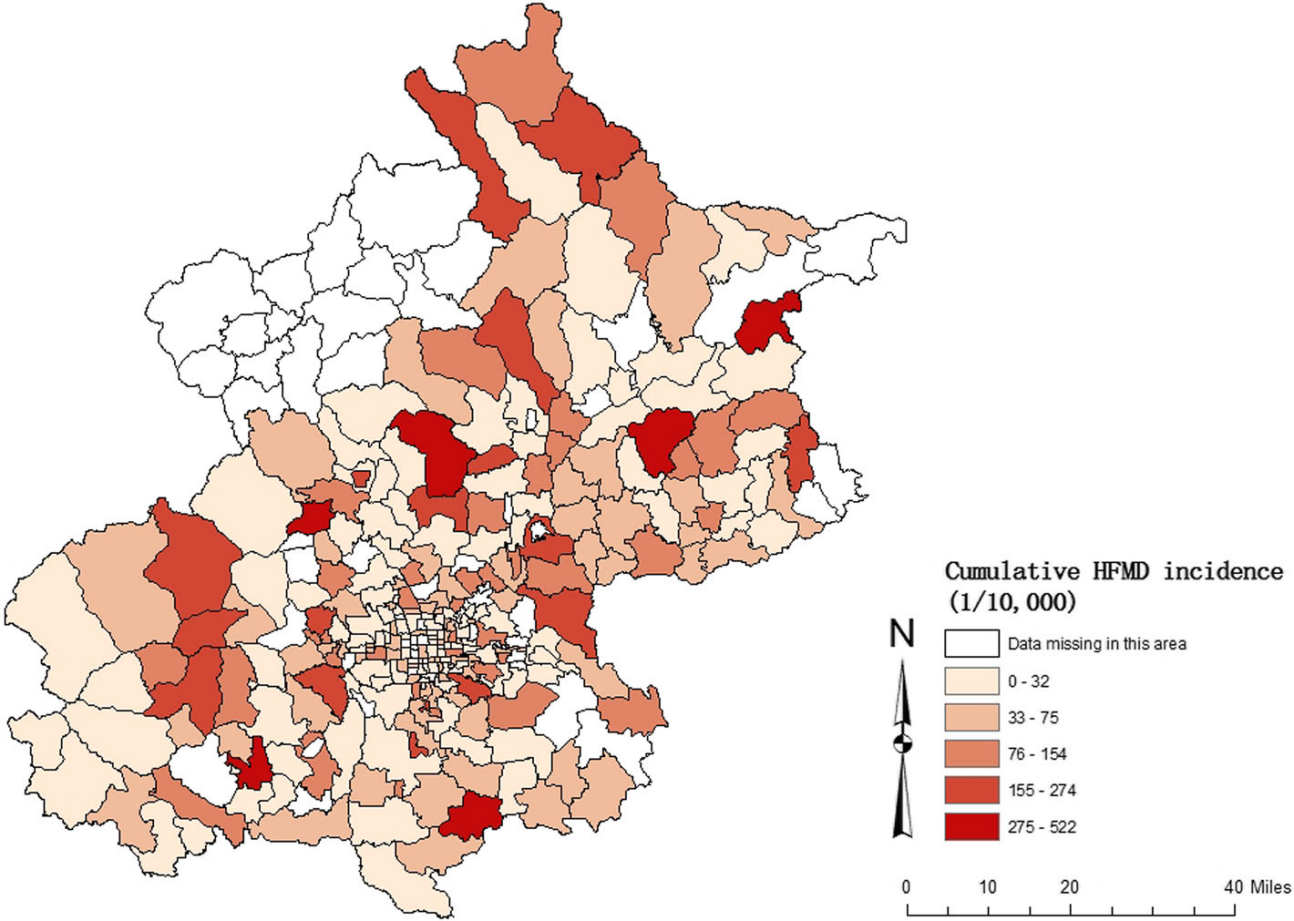
Spatial distribution of cumulative HFMD incidence in Beijing, 2010–2012

Figure 2 shows the spatial distribution of cumulative incidence of HFMD at the township level during the study period. The raw incidence was 231.5 cases per 100,000 people in 2010, 152.8 cases per 100,000 in 2011 and 186.2 cases per 100,000 people in 2012, with an average annual incidence of 190.2 for these 3 years. Most cases were reported in central and southern regions (Additional file 2: Figure S1).

### Temporal variability of meteorological variables

**Fig. 3:**
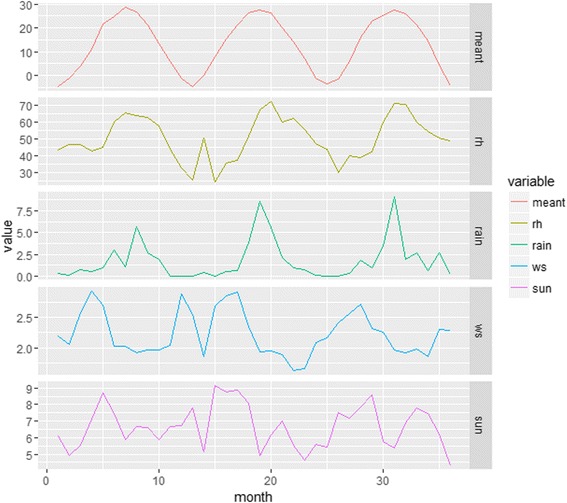
Monthly time-series for the meteorological factors in Beijing, 2010–2012. (meant, mean temperature (°C); rh, relative humidity (%); rain, precipitation (mm); ws, wind velocity (m/s); sun, sunshine (hour))

The monthly time-series of the meteorological factors is illustrated in Fig. 3, with summary statistics provided in Table 4. Mean temperature had a seasonal pattern with peaks in July and August (summer in Beijing). Relative humidity and precipitation had similar patterns, peaking just after temperature (late summer). Wind velocity did not show a regular pattern and sunshine hours were longest in spring and autumn.

### Statistical models

A high level of correlation was found between mean temperature and both maximum temperature (*r* = 0.987) and minimum temperature (*r* = 0.981). Mean temperature was included in the subsequent model as a temperature index. Air pressure was also highly correlated with mean temperature (*r* = − 0.869) and was excluded. Thus five meteorological factors (mean temperature, relative humidity, precipitation, sunshine hours and wind velocity) were chosen for inclusion in the regression models for further analysis. Table 3 summarizes the DIC values for the three models. The spatio-temporal model had the lowest DIC, indicating it was the best performing model. The spatio-temporal model was therefore selected as the final model, with meteorological factors included as covariates.

**Table 3:** The Deviance Information Criterion values for Bayesian models of HFMD incidence

Model	DIC	
	Without covariates	With covariates
Non-spatial model	55,654.9	–
Spatial model	55,579.0	–
Spatial-temporal model	52,352.4	52,254.8

Table 4 presented the association between meteorological factors and HFMD incidence, which indicated that mean temperature, relative humidity, wind velocity and sunshine hours were all positively associated with HFMD risk, whereas precipitation was not significantly associated with HFMD risk. The effect of wind velocity, sunshine hours, mean temperature and relative humidity was significant with RRs of 3.30 (95%CI: 2.37, 4.60), 1.20 (95%CI: 1.02, 1.40), 1.06 (95%CI: 1.03, 1.10) and 1.05 (95%CI: 1.01, 1.08) per unit increase, respectively.

**Table 4:** Bayesian Poisson regression model of the association between meteorological factors and HFMD in Beijing, 2010–2012

Variable	Mean	Range	Coefficient (95%CI)	Relative Risk (95%CI)
Mean temperature (°C)	12.94	−4.78-28.61	0.06(0.03,0.09)	1.06(1.03,1.10)
Relative humidity (%)	50.35	24.77–71.90	0.05(0.01,0.08)	1.05(1.01,1.08)
Precipitation (mm)	1.79	0–9.16	0.03(−0.06,0.12)	1.03(0.94,1.12)
Wind velocity (m/s)	2.23	1.62–2.93	1.19(0.86,1.53)	3.30(2.37,4.60)
Sunshine (hour)	6.67	4.32–9.14	0.18(0.02,0.34)	1.20(1.02,1.40)
Overall Intercept			−9.53(−11.37,-7.68)	

**Fig. 4:**
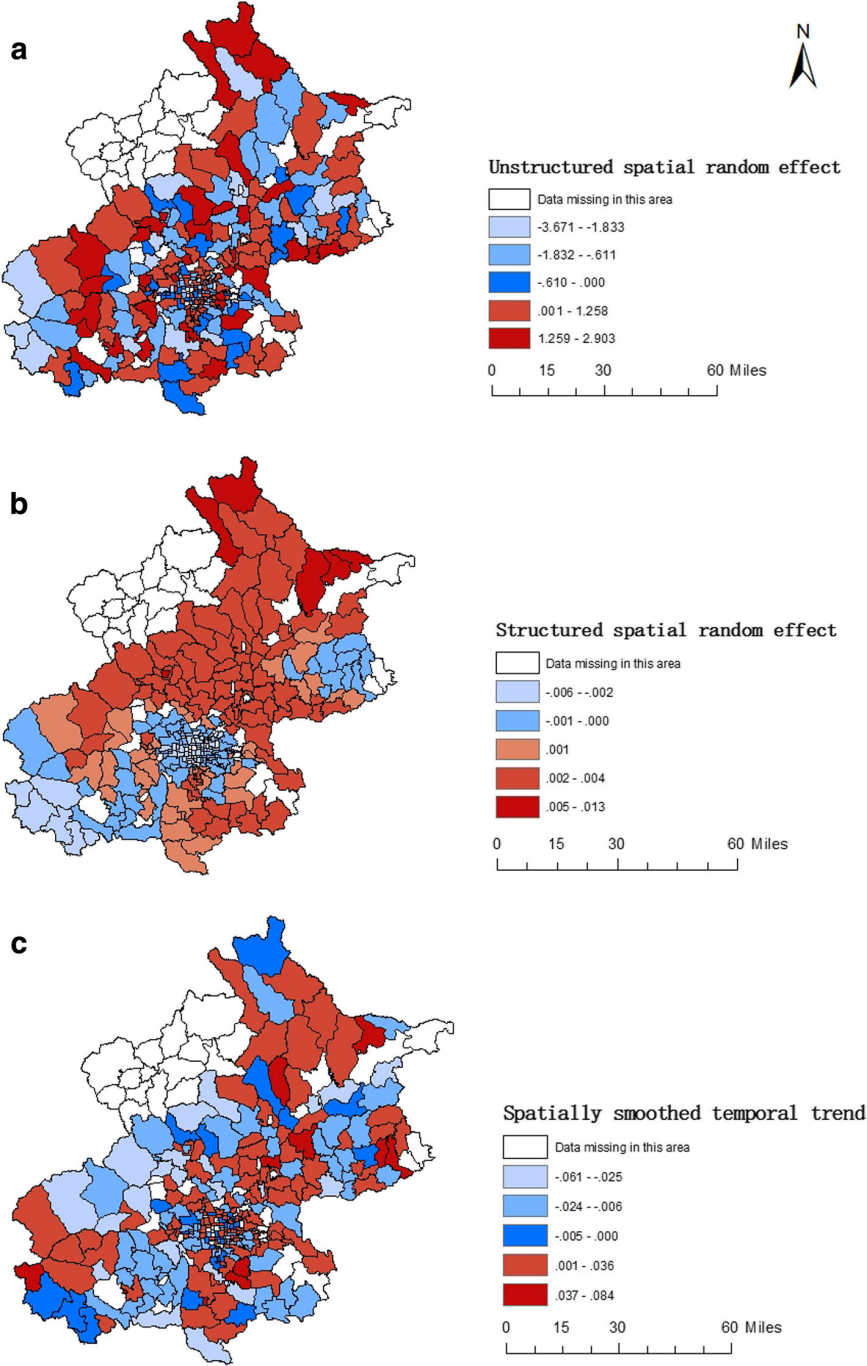
Unstructured, structured spatial random effect and spatially smoothed temporal trend for HFMD in Beijing, 2010–2012

Figure 4 shows maps of the township-level mean for each of the variance components of the model (A – unstructured spatial random effect; B – structured spatial random effect; and C – spatially smoothed temporal trend). Clear clusters of low relative risk were present in central, northeast and southwest Beijing, with clusters of high relative risk evident in the north and southeast. The spatially smoothed temporal trend did not show a very clear pattern, with small clusters of higher and lower than average trends interspersed throughout Beijing.

## Discussion

There were 114,777 HFMD cases from 2010 to 2012, with an average incidence of 190.2 cases per 100,000 people, which was higher than some other provinces in China like Sichuan (43.7/100,000, 2008–2013) [31], Shandong (104.4/100,000, 2008–2012) [23], Jiangsu (126.3/100,000, 2009–2013) [24], and Guangdong (167.8/100,000, 2008–2011) [32], but was lower than Guangxi (298.3/100,000, 2008–2013) [21]. The total incidence in males was higher than that in females, which was consistent with previous studies [23, 24], suggesting the susceptibility of males. Most cases were concentrated in children from 1 to 4 years old, which were the main target groups for the surveillance and control of HFMD in Beijing.

From 2010 to 2012, there was an oscillating inter-annual pattern with peaks at the start and towards the end of the study period in HFMD incidence. The peaks of HFMD cases were in May to July every year, which corresponds to early summer in Beijing. There was another lower peak occurring in the autumn of 2011. Previous studies in Singapore and Malaysia have shown seasonal outbreaks of HFMD between March and May [22], whereas in Japan, outbreaks typically occur during the summer months [33]. In China, single seasonal peaks have been shown to appear between April and August in Shandong province, as well as Henan province, both also located in North China [23]. Double peaks have been observed in some southern districts of China, usually in the warm months of spring and autumn [34, 35].

The areas of Beijing with high HFMD incidence were distributed in districts circumjacent to central Beijing like Mentougou, Fangshan, Changping, Daxing, Tongzhou and north Huairou district (Fig. 2). The spatial random effect (Fig. 4) represented the residual spatial clustering after accounting for the meteorological variables [36]. Socio-economic levels, medical and health facility access, surveillance and control capacities of HFMD in different districts might be potential factors influencing this residual variation, which are more amenable to control than meteorological factors. Thus, clusters of high residual risk should be the focus of greater attention in the prevention and control of HFMD with measures like reinforcing community health education and improving health care levels of nurseries.

Mean temperature, relative humidity, wind velocity and sunshine hours were positively associated with HFMD incidence. Temperature and relative humidity were found to be associated with HFMD in previous studies [14, 33, 37]. There are two main ways that meteorological factors can influence HFMD: by affecting the external environment to change the biological activity, propagation and transmission of pathogen; and by impacting on human behavior [38–40]. During warmer months, communal physical activity among children and adolescents increases, which may promote the risk of contact transmission of HFMD [41–43]. A study in Shandong province [44] revealed a strong association between HFMD incidence and wind speed as shown in our study. They suggested that wind can promote air pollutants like particulate matter carrying enterovirus and thus accelerate the spread of HFMD [38]. As a major atmospheric pollutant, fine particulate matter (PM_2.5_, defined as particle less than 2.5 mm aerodynamic diameter) has a small size and a relatively large surface area, which makes it easy to absorb viruses in the air. Especially considering the high levels of PM_2.5_ pollution in Beijing [45, 46], it is plausible that high wind velocity is a risk factor for the spread of HFMD. On the other hand, children spend more time with indoor activities in confined spaces during windy periods, which would increase the chances of EV transmission, a possible indirect explanation supporting the association of increased transmission with windy conditions. Longer sunshine hours was also found to be a risk factor in our study, which was different from studies in other places [23, 24]. Sunlight could promote virus replication or inactivate human virus to some extent [47–49]. It is also possible that children engage more in outside activities on sunny days, thus increasing contact among children. Precipitation was not significantly associated with HFMD incidence in this study, which was consistent with the study conducted in Shandong Province [23]. A study in Hefei [50] of China found that extreme precipitation (90th percentile of precipitation as the analytical cut-off point) was significantly associated with childhood HFMD. Beijing is a temperate city with lower mean monthly precipitation (54.9 mm) than Hefei (130.8 mm), which has a subtropical monsoon humid climate The threshold effect of precipitation on HFMD might be the reason for inconsistent conclusions [50].

There were previous studies conducted in Beijing investigating spatio-temporal patterns of HFMD and the effects of meteorological factors on this disease. Additional file 1: Table S1 showed the detailed comparison of these studies with ours. Wang et al. [18] used spatial filtering combined with scan statistics methods to detect HFMD clusters in Beijing from 2008 to 2012, finding that the most likely space-time cluster was located in the southwest of Beijing. Dong et al. [38] used geographically weighted regression model to explore the seasonal influence of weather factors on incidents of HFMD from 2008 to 2011 in Beijing. Our study considered spatial and temporal variability of all the variables in Bayesian CAR model, which could quantify spatio-temporal variation and identify the effects of meteorological factors at the same time. Compared with the study conducted by Dong et al., we found that sunshine hours was also positively associated with HFMD incidence. The effects of mean temperature and wind velocity were consistent while relative humidity and precipitation showed different effect estimates. The threshold effect of precipitation on HFMD incidence might be the reason for the discordant conclusions.

Demographic and socio-economic characteristics which include child population density, Gross Domestic Product per capita, number of health agencies, proportion of children in nursery, and proportion of children in primary school might be confounders affecting the incidence of HFMD. Additional file 3: Figure S2, Additional file 4: Figure S3, Additional file 5: Figure S4, Additional file 6: Figure S5, Additional file 7: Figure S6 were the spatial distributions of these factors, which did not show obvious association with HFMD clusters. However, data of the demographic and socio-economic characteristics that we obtained from Beijing Area Statistics Yearbook are in district level for now and further statistical analysis considering these factors in higher resolution should be conducted if available.

In terms of study limitations, the meteorological data were obtained from one monitoring center, which was assumed to represent the meteorological conditions of the whole city. It would be preferable to incorporate spatial variation in the meteorological variables. However, Beijing has a relatively small area, and we do not expect as much spatial variability between townships as temporal variability across months and years. Secondly, the time-series was short (3 years) and to quantify seasonal and interannual variability and associated factors it would be better to analyze data from a much longer time period. Finally, this was an ecological study and it is important to note that measured associations were only valid at the level of the township and cannot be applied to individuals, or different levels of spatial aggregation [23].

## Conclusions

In this study, we detected the spatial and temporal distribution of HFMD and its association with meteorological factors by using Bayesian spatiotemporal regression models in Beijing, China. The incidence of HFMD cases had a seasonal pattern in Beijing and the peaks were often in warm months. Certain districts with high HFMD incidence should be the focus of greater attention. Meteorological factors like temperature, relative humidity, wind velocity and sunshine hours might be the driving factors for the spatiotemporal dynamics of HFMD, and could be considered as targets for meteorological monitoring of HFMD risk.

## Additional files

Additional file 1: **Table S1.** Comparison of studies conducted in Beijing investigating spatio-temporal patterns of HFMD and the effects of meteorological factors. (DOCX 16 kb)

Additional file 2: **Figure S1.** Spatial distribution of cumulative HFMD cases in Beijing, 2010–2012. (JPEG 289 kb)

Additional file 3: **Figure S2.** Spatial distribution of average child population density in Beijing, 2010–2012. (JPEG 128 kb)

Additional file 4: **Figure S3.** Spatial distribution of Gross Domestic Product per capita (billion) in Beijing, 2012. (JPEG 134 kb)

Additional file 5: **Figure S4.** Spatial distribution of health agency counts (per square kilometers) in Beijing, 2012. (JPEG 141 kb)

Additional file 6: **Figure S5.** Spatial distribution of proportion of children in nursery in Beijing, 2012. (JPEG 137 kb)

Additional file 7: **Figure S6.** Spatial distribution of proportion of children in primary school in Beijing, 2012. (JPEG 123 kb)

## Abbreviations

CAR: Bayesian conditional auto regressive
CDC: Center for Disease Prevention and Control
CISDCP: China Information System for Disease Control and Prevention
Cox A16: Coxsackievirus A16
DIC: Deviance information criterion
EV71: Enterovirus 71
HFMD: Hand-foot-mouth disease
MCMC: Markov chain Monte Carlo
SMRs: Standardized morbidity ratios
