# Supplementary material for: Spatio-temporal analysis of the relationship between meteorological factors and hand-foot-mouth disease in Beijing, China
Source: BMC Infect Dis. 2018 Apr 3;18:158. doi: 10.1186/s12879-018-3071-3 (PMC5883540; doi:10.1186/s12879-018-3071-3)
Supplement: Supplementary file 1 — Table S1. Comparison of studies conducted in Beijing investigating spatio-temporal patterns of HFMD and the effects of meteorological factors. (DOCX 16 kb) [file 12879_2018_3071_MOESM1_ESM.docx]

**Table S1 Comparison of studies conducted in Beijing investigating spatio-temporal patterns of HFMD and the effects of meteorological factors.**

| **Study period** | **Model** | **Incidence peaks** | **Spatial patterns** | **Associated meteorological factors** | **Correlation with HFMD** | **Reference** |
| --- | --- | --- | --- | --- | --- | --- |
| 2010-2012 | Bayesian conditional auto regressive model | May to July | Clusters of high relative risk were in the north and southeast Beijing. | mean temperature | positive | This study |
|  |  |  |  | relative humidity | positive |  |
|  |  |  |  | wind velocity | greatest effect; positive |  |
|  |  |  |  | sunshine | positive |  |
|  |  |  |  | precipitation | non-significant |  |
| 2008-2011 | Geographically weighted regression model | May to Oct | - | average temperature | greatest effect; positive | Dong et al.* |
|  |  |  |  | average wind speed | positive |  |
|  |  |  |  | total precipitation | positive in the summer, negative in the winter |  |
|  |  |  |  | average relative humidity | negative |  |
| 2008-2012 | Local Indicators of Spatial Association | April to July | Higher relative risk areas were located in the urban-rural transition zones. | - | - | Wang et al.** |
|  | Spatial filtering combined with scan statistics methods |  | The most likely cluster was located in the southwest of Beijing. |  |  |  |

* Dong, W., et al., *The Effects of Weather Factors on Hand, Foot and Mouth Disease in Beijing.* Sci Rep, 2016. **6**: p. 19247.

** Wang, J., et al., *Epidemiological analysis, detection, and comparison of space-time patterns of Beijing hand-foot-mouth disease (2008-2012).* PLoS One, 2014. **9**(3): p. e92745.
