# Supplementary figures and images for: Spatio-temporal analysis of the relationship between meteorological factors and hand-foot-mouth disease in Beijing, China
Source: BMC Infect Dis. 2018 Apr 3;18:158. doi: 10.1186/s12879-018-3071-3 (PMC5883540; doi:10.1186/s12879-018-3071-3)

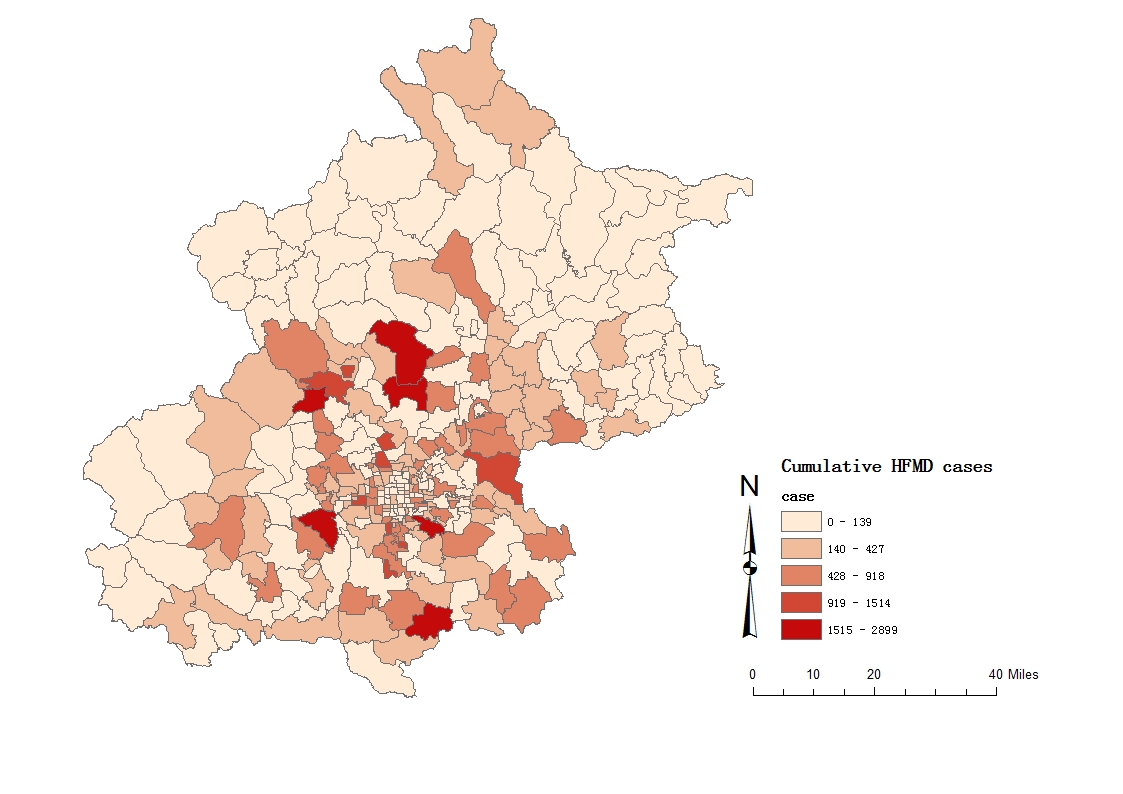

Supplement: Supplementary file 2 — Figure S1. Spatial distribution of cumulative HFMD cases in Beijing, 2010–2012. (JPEG 289 kb) [file 12879_2018_3071_MOESM2_ESM.jpg]

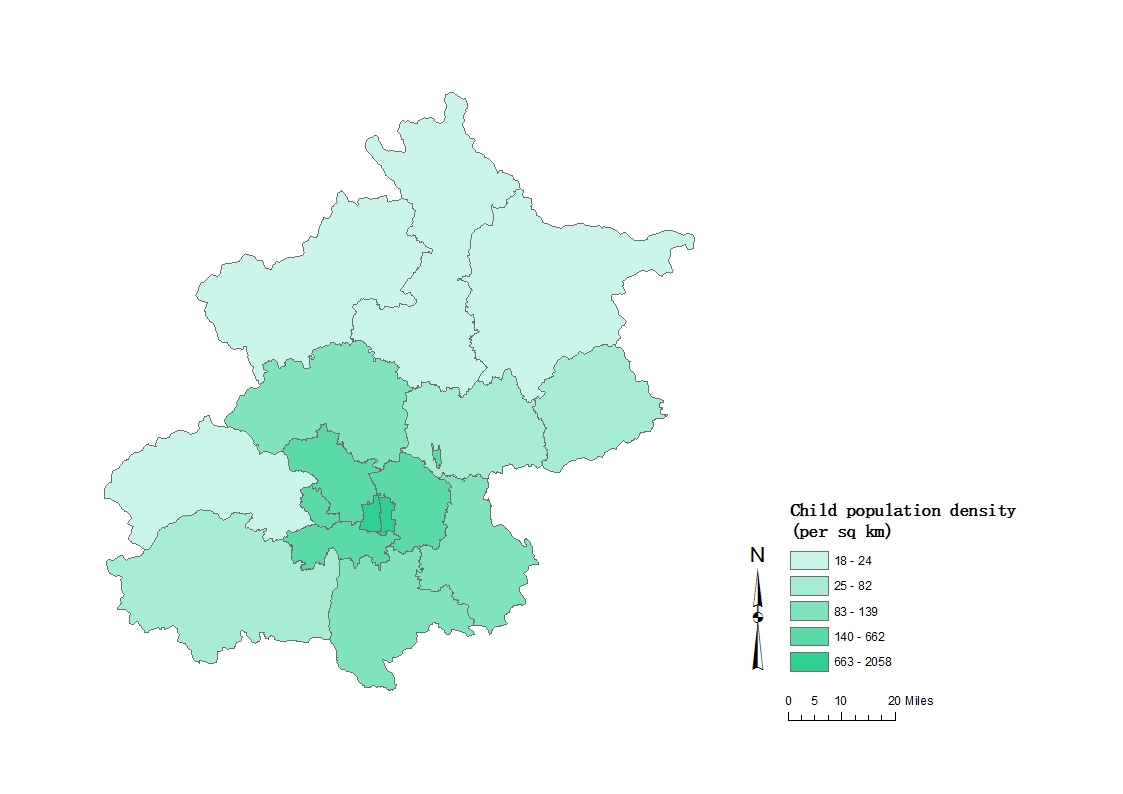

Supplement: Supplementary file 3 — Figure S2. Spatial distribution of average child population density in Beijing, 2010–2012. (JPEG 128 kb) [file 12879_2018_3071_MOESM3_ESM.jpg]

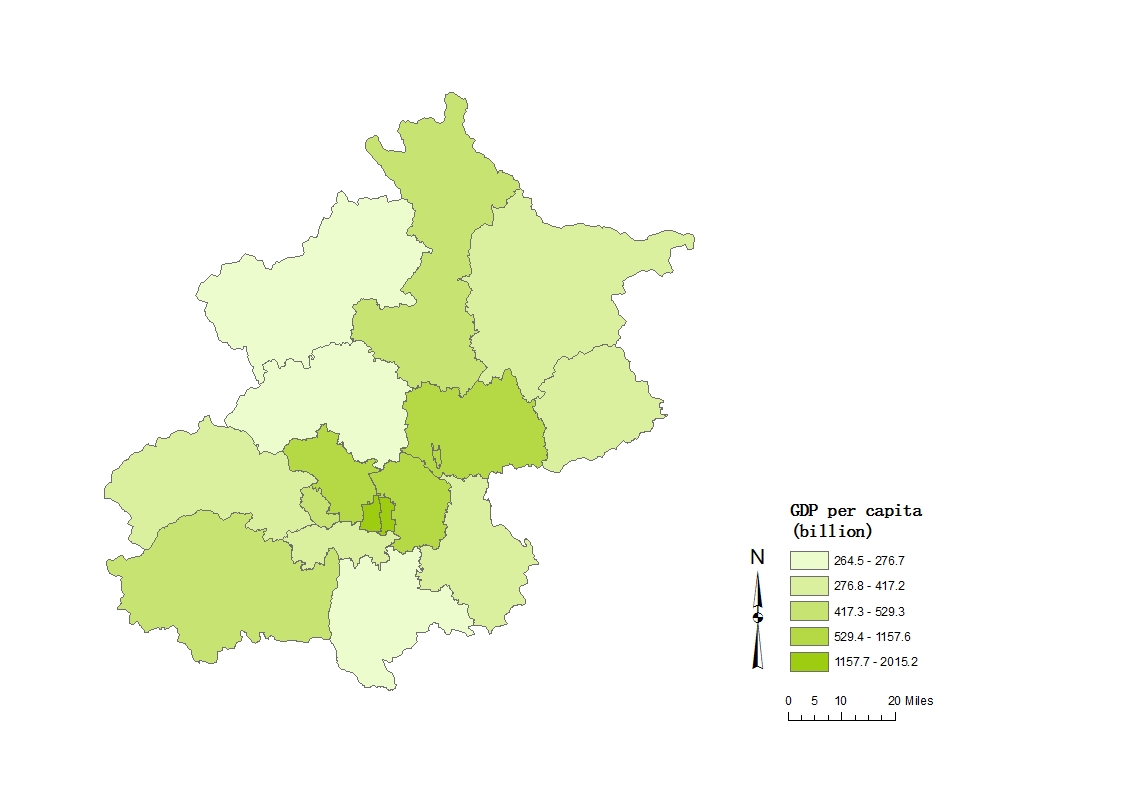

Supplement: Supplementary file 4 — Figure S3. Spatial distribution of Gross Domestic Product per capita (billion) in Beijing, 2012. (JPEG 134 kb) [file 12879_2018_3071_MOESM4_ESM.jpg]

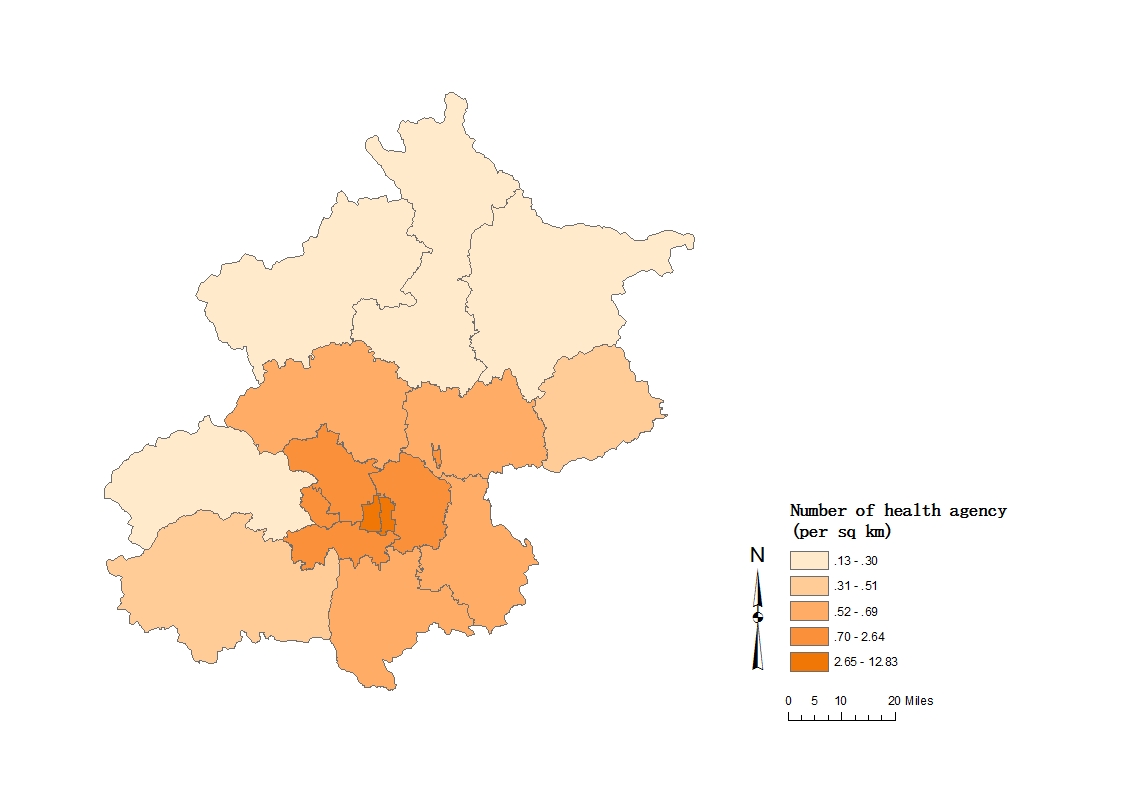

Supplement: Supplementary file 5 — Figure S4. Spatial distribution of health agency counts (per square kilometers) in Beijing, 2012. (JPEG 141 kb) [file 12879_2018_3071_MOESM5_ESM.jpg]

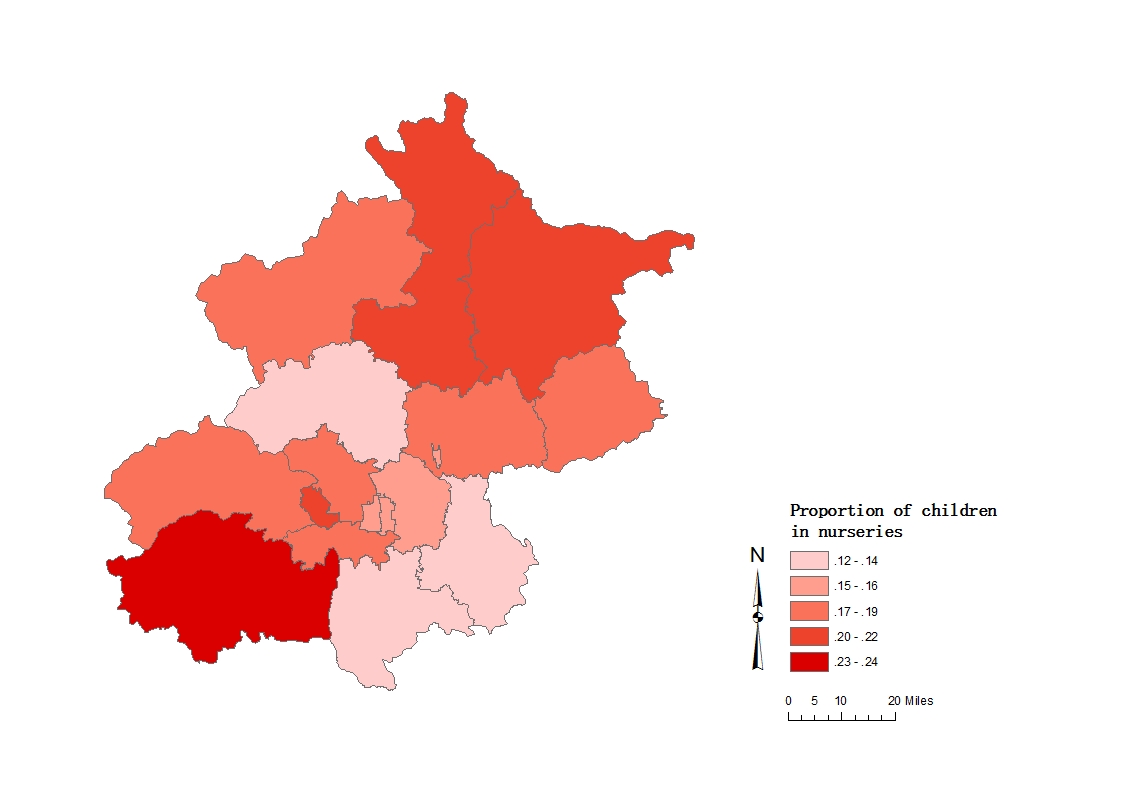

Supplement: Supplementary file 6 — Figure S5. Spatial distribution of proportion of children in nursery in Beijing, 2012. (JPEG 137 kb) [file 12879_2018_3071_MOESM6_ESM.jpg]

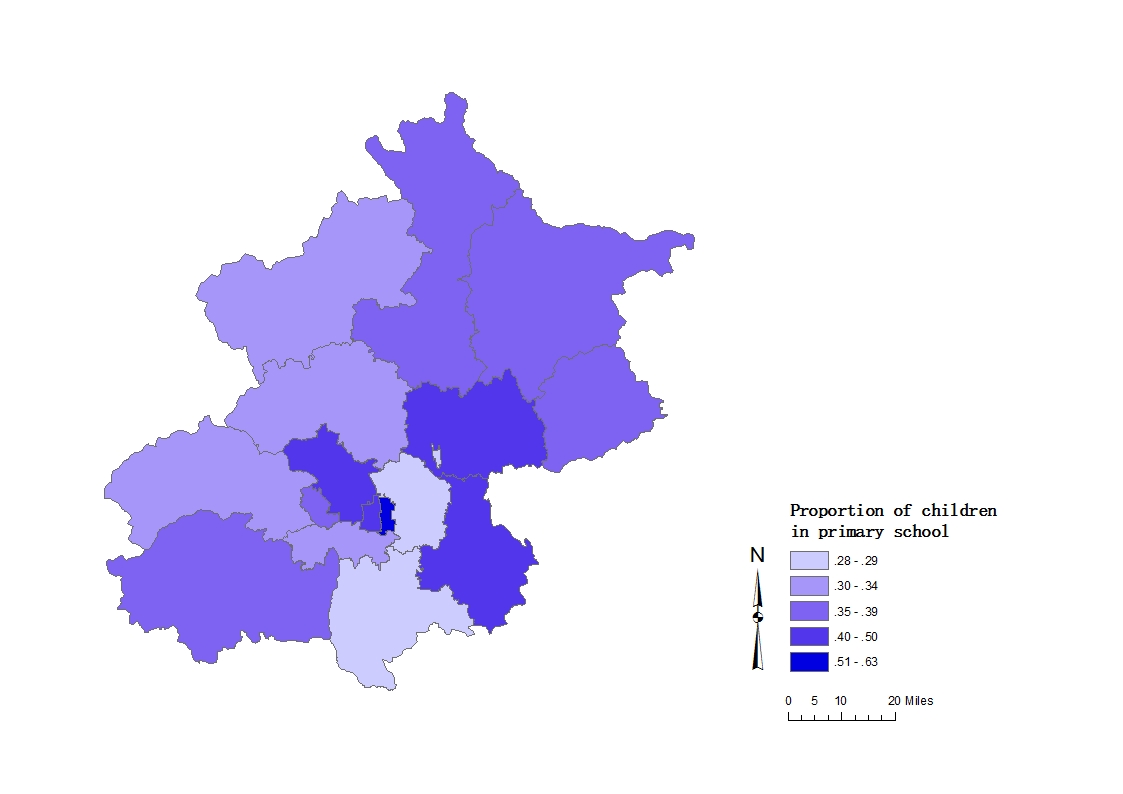

Supplement: Supplementary file 7 — Figure S6. Spatial distribution of proportion of children in primary school in Beijing, 2012. (JPEG 123 kb) [file 12879_2018_3071_MOESM7_ESM.jpg]
